# Force sensitivity of multilayer graphene optomechanical devices

**DOI:** 10.1038/ncomms12496

**Published:** 2016-08-09

**Authors:** P. Weber, J. Güttinger, A. Noury, J. Vergara-Cruz, A. Bachtold

**Affiliations:** 1 ICFO-Institut de Ciencies Fotoniques, The Barcelona Institute of Science and Technology, Castelldefels (Barcelona) 08860, Spain

## Abstract

Mechanical resonators based on low-dimensional materials are promising for force and mass sensing experiments. The force sensitivity in these ultra-light resonators is often limited by the imprecision in the measurement of the vibrations, the fluctuations of the mechanical resonant frequency and the heating induced by the measurement. Here, we strongly couple multilayer graphene resonators to superconducting cavities in order to achieve a displacement sensitivity of 1.3 fm Hz^−1/2^. This coupling also allows us to damp the resonator to an average phonon occupation of 7.2. Our best force sensitivity, 390 zN Hz^−1/2^ with a bandwidth of 200 Hz, is achieved by balancing measurement imprecision, optomechanical damping, and measurement-induced heating. Our results hold promise for studying the quantum capacitance of graphene, its magnetization, and the electron and nuclear spins of molecules adsorbed on its surface.

## Abstract (web summary)

Graphene is a promising material for the design of mechanical resonators. Here, the authors fabricate a multilayer graphene resonator coupled to a superconducting cavity, to achieve efficient readout of mechanical vibrations and quantitatively investigate its force sensing performance.

## Graphical abstract

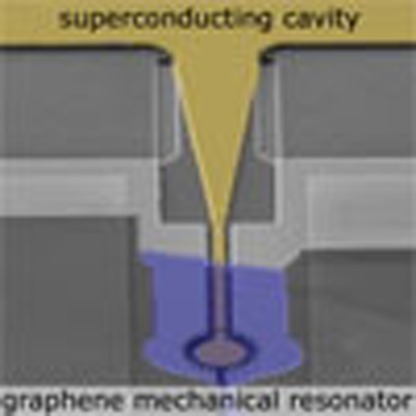

Considerable effort has been devoted to developing mechanical resonators based on low-dimensional materials, such as carbon nanotubes^1^,^2^,^3^,^4^,^5^,^6^,^7^,^8^,^9^,^10^,^11^,^12^, semiconducting nanowires^13^,^14^,^15^,^16^,^17^,^18^,^19^,^20^,^21^,^22^, graphene^23^,^24^,^25^,^26^,^27^,^28^,^29^ and monolayer semiconductors^30^,^31^,^32^. The specificity of these resonators is their small size and their ultra-low mass, which enables sensing of force and mass with unprecedented sensitivities^7^,^10^. Such high-precision sensing capabilities hold promise for studying physical phenomena in new regimes that have not been explored thus far, for instance, in spin physics^33^, quantum electron transport^34^,^35^, light-matter interaction^19^ and surface science^36^,^37^. However, the transduction of the mechanical vibrations of nanoscale mechanical systems into a measurable electrical or optical output signal is challenging. As a result, force and mass sensing is often limited by the imprecision in the measurement of the vibrations, and cannot reach the fundamental limit imposed by thermo-mechanical noise.

A powerful method to obtain efficient electrical readout of small resonators is to amplify the interaction between mechanical vibrations and the readout field using a superconducting microwave cavity^27^,^28^,^29^. Increasing the field in the cavity improves the readout sensitivity and eventually leads to dynamical back-action on the thermo-mechanical noise. This effect has been studied intensively on comparatively large micro-fabricated resonators, resulting for instance in enhanced optomechanical damping^38^,^39^, ground-state cooling of mechanical vibrations^40^,^41^ and displacement imprecision below the standard quantum limit^42^,^43^. Another phenomenon often observed when detecting and manipulating the motion of mechanical resonators is the induced heating that can occur through Joule dissipation and optical adsorption^28^,^44^. Heating is especially prominent in tiny mechanical resonators because of their small heat capacity. An additional difficulty in characterizing mechanical vibrations is related to the fluctuations of the mechanical resonant frequency, also called frequency noise, which are particularly sizable in small resonators endowed with high-quality factors *Q*^10^.

Here we study the force sensitivity of multilayer graphene mechanical resonators coupled to superconducting cavities. In particular, we quantify how the force sensitivity is affected by dynamical back-action, Joule heating and frequency noise upon increasing the number of pump photons inside the cavity. We demonstrate a force sensitivity of 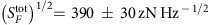, of which ≈50% arises from thermo-mechanical noise and ≈50% from measurement imprecision. The force sensitivity tends to be limited by measurement imprecision and frequency noise at low pump power, and by optomechanical damping and Joule heating at high pump power.

## Results

### Thermal force noise and imprecision force noise

**Figure 1:**
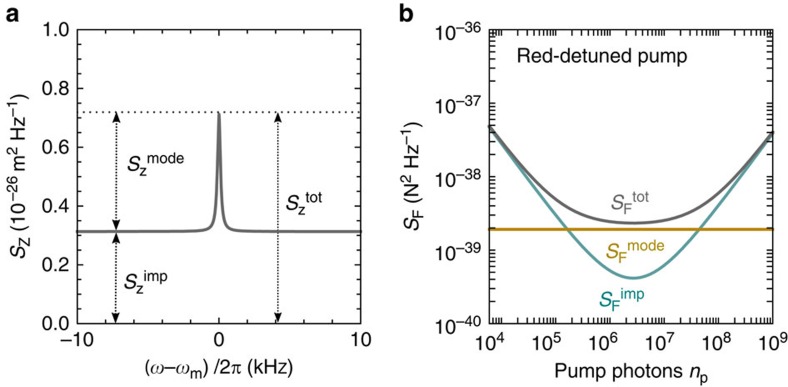
Mechanical displacement and force sensitivity. (**a**) Mechanical displacement spectrum *S*_*z*_ close to the mechanical resonance frequency *ω*_m_/2*π*. The total displacement spectral density 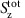 at *ω*_m_ is the sum of the displacement noise 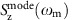 and the displacement imprecision 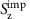. (**b**) Corresponding force sensitivity 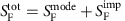 (dark grey). The individual components are the thermal force noise 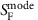 (dark yellow) and the imprecision force noise 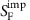 (turquoise), given by equations (1) and (2), respectively. The quantum back-action noise is neglected for simplicity. For the plots most of the parameters are those of device B, but we estimate the mass assuming that the graphene flake is a single layer. Further we choose *n*_add_=0.5, *T*_bath_=0.015 K, and *n*_p_=2·10^5^ in **a** (see text).

A fundamental limit of force sensing is set by the thermo-mechanical noise of the eigenmode that is measured. According to the fluctuation-dissipation theorem, the associated thermal force noise is white and is quantified by


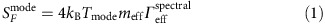


where *T*_mode_ is the temperature of the mechanical eigenmode, and *m*_eff_ is its effective mass^8^,^45^. This force noise is transduced into a mechanical resonance with line width 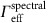 and height 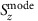 in the displacement spectrum (Fig. 1). Importantly, equation (1) shows that the low mass of graphene decreases the size of the thermo-mechanical force noise. However, a drawback of tiny resonators with high *Q*-factors is their tendency to feature sizable frequency noise that broadens the resonance and, therefore, increases the size of the force noise^10^,^46^.

Measuring mechanical vibrations with high accuracy is key to resolving small forces, since the imprecision in the measurement contributes to the force sensitivity. The force sensitivity 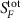 is given by the sum of the thermal force noise 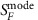 and the imprecision force noise 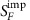, where the latter is the result of the white noise background with strength 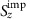 in the displacement spectrum (Fig. 1a). The challenge with mechanical resonators based on low-dimensional systems is to reach the limit 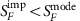. When detecting the motion of graphene resonators with microwave cavities, one typically operates in the resolved sideband limit^27^,^28^,^29^, where the cavity decay rate *κ* is significantly smaller than the mechanical resonance frequency *ω*_m_. This is interesting for force sensing, because pumping on the red sideband allows to enhance the mechanical damping rate by *Γ*_opt_, and therefore to reduce the harmful effect of frequency noise, as we will discuss below. In addition, this allows to increase the measurement bandwidth, as is often done in magnetic resonance force microscopy experiments^33^ while keeping 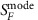 constant. The drawback of red sideband pumping compared to pumping at the cavity resonant frequency is an increased imprecision force noise at high pump powers. In the red-detuned pump regime, the measurement imprecision contributes to the force sensitivity by the amount


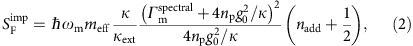


with *κ*_ext_ the external coupling rate of the cavity, *n*_add_ the noise added by the amplifier chain at the output of the device, *Γ*_m_^spectral^ the intrinsic line width of the resonator, *n*_p_ the number of pump photons in the cavity, and *g*_0_ the single-photon optomechanical coupling. Figure 1b shows the pump power dependence of the force sensitivity 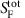 expected in the absence of Joule heating and frequency noise. The increase of 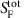 at high *n*_p_ is due to the dynamical back-action, which enhances the mechanical line width by *Γ*_opt_=4*n*_p_*g*_0_^2^/*κ*.

### Device characterization

**Figure 2:**
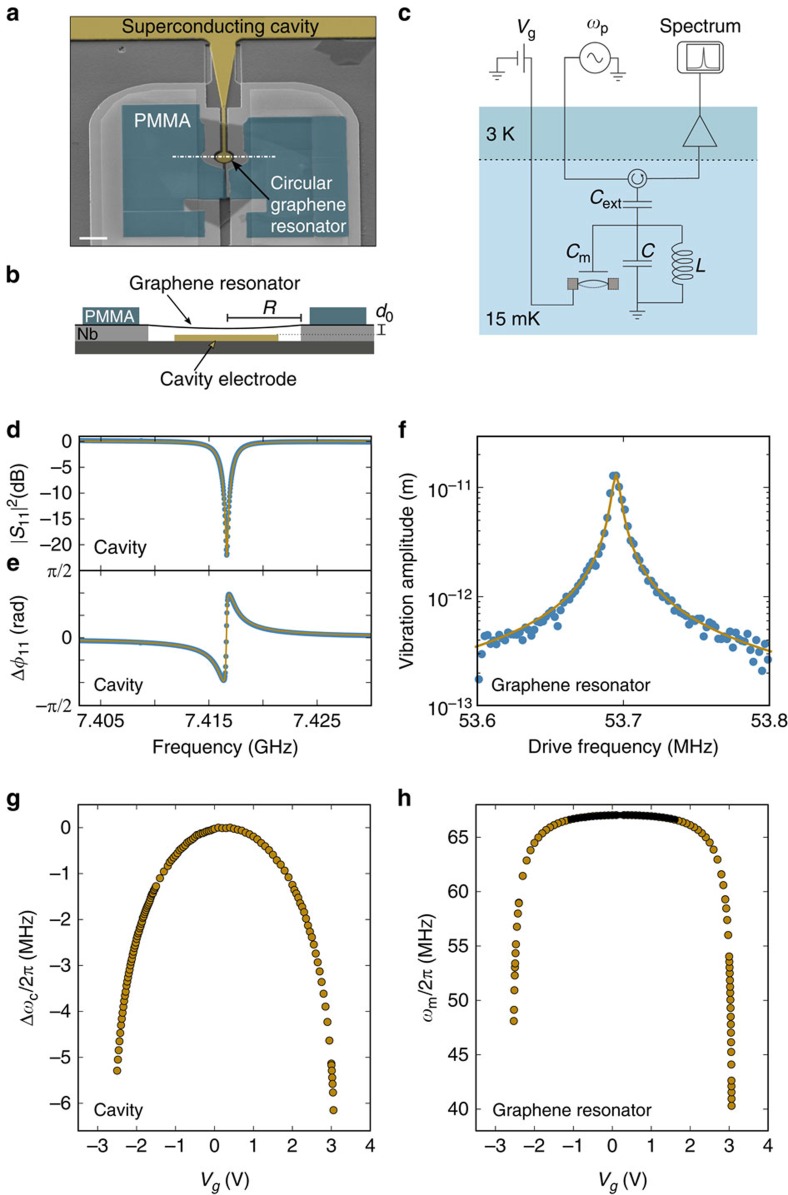
Device and characterization. (**a**) False-colour image of the device. The cavity is coloured in dark yellow. The graphene flake is clamped in between niobium support electrodes (grey) and cross-linked poly(methyl metracylate) (turquoise). The scale bar is 5 μm. (**b**) Schematic cross-section of the graphene resonator along the white dashed dotted line in **a**. (**c**) Schematic of the measurement circuit. The graphene mechanical resonator is coupled to the superconducting LC cavity through the capacitance *C*_m_. The separation *d* between the suspended graphene flake and the cavity counter electrode is controlled by the constant voltage *V*_g_. The cavity is pumped with a pump tone at *ω*_p_ and the output signal is amplified at 3 K. (**d**) Reflection coefficient |*S*_11_|^2^ and (**e**) reflected phase Δ*φ*_11_ of the superconducting cavity of device A at *V*_g_=3.002 V. The dark yellow lines are fits to the data using *κ*_int_/2*π*=950 kHz and *κ*_ext_/2*π*=850 kHz using equation (7) (see Methods). (**f**) Driven vibration amplitude of the graphene resonator of device A as a function of drive frequency. The driving voltage is 22 nV and *V*_g_=3.002 V. The dark yellow line is a lorentzian fit to the data. (**g**) Resonant frequency *ω*_c_/2*π* of the superconducting cavity as a function of *V*_g_. (**h**) Resonant frequency *ω*_m_/2*π* of the graphene resonator as a function of *V*_g_. The black line is the *V*_g_ dependence of *ω*_m_ expected from electrostatic softening (see Supplementary Note 1).

Our devices consist of a suspended graphene mechanical resonator capacitively coupled to a superconducting niobium (Nb) cavity (Fig. 2a–c). The graphene resonators are circular with a radius of *R*≈1.6 μm. Here we present data of two devices. The graphene resonator of device A has a thickness of approximately 25 layers, and the one of device B 5–6 layers. This corresponds respectively to an effective mass of *m*_eff_=(4.1±0.8)·10^−17^ kg and (9.6±0.8)·10^−18^ kg. The uncertainty results from extracting the mass with different methods including optical contrast measurements, thickness measurements with atomic force microscopy and the measured electrostatic softening of the mechanical resonators (see Supplementary Note 1 and Supplementary Equation 2). The fundamental mode of devices A and B vibrates at *ω*_*m*_/2*π*=67 MHz and *ω*_m_/2*π*=46 MHz at *V*_g_=0 V, respectively. Here *V*_g_ is the constant voltage applied between the graphene flake and the superconducting cavity. In order to improve the attachment of the graphene flake to its support, we clamp it between cross-linked poly(methyl metracylate) and the contact electrodes; the detailed fabrication is described elsewhere^29^. The separation between the graphene resonator and the cavity counter electrode at *V*_g_=0 V is assumed to be equal to the hole depth, which is typically *d*_0_≈85 nm in our devices as measured with atomic force microscopy. Varying *V*_g_ allows us to tune the separation between the graphene resonator and the cavity counter electrode^24^,^29^,^47^,^48^,^49^, modifying the graphene-cavity capacitance, the cavity frequency *ω*_c_ and *ω*_m_ (Fig. 2g,h). The superconducting cavity is a coplanar waveguide resonating at about *ω*_c_/2*π*=7.4 GHz. We choose a single-port, quarter wavelength, reflection geometry, so that the cavity is connected to ground on one end, allowing to apply a well-defined constant voltage between the cavity and the graphene flake. The other end of the cavity is coupled to a transmission line via a capacitor *C*_ext_ with a coupling rate *κ*_ext_=2*π* × 850 kHz for device A; the total cavity decay rate is *κ*=*κ*_ext_+*κ*_int_=2*π* × 1.8 MHz (see Methods). Here *κ*_int_ accounts for the internal energy loss.

We detect the vibrations of the graphene resonator with high precision by pumping the cavity with an electromagnetic field, and probing its mechanical sideband. This sideband is generated by the capacitive modulation of the pump field at frequency *ω*_p_/2*π* by the graphene vibrations at *ω*_m_/2*π*. We usually set *ω*_p_=*ω*_c_−*ω*_m_ and probe the electromagnetic field that exits the cavity at *ω*_c_. We measure the device at the cryostat base temperature of 15 mK if not stated otherwise. The cavity output field is amplified with a high electron-mobility-transistor mounted at the 3 K stage of the cryostat. Mechanical noise spectra are detected with a spectrum analyser at room temperature. For a detailed description of the measurement set-up, see Supplementary Fig. 1 and Supplementary Note 2. In addition, we perform ring-down measurements to determine the mechanical dissipation rate 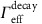 of the graphene resonator. Spectral measurements are not suitable for quantifying reliably 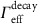 because of the potentially substantial frequency noise of graphene resonators.

**Figure 3:**
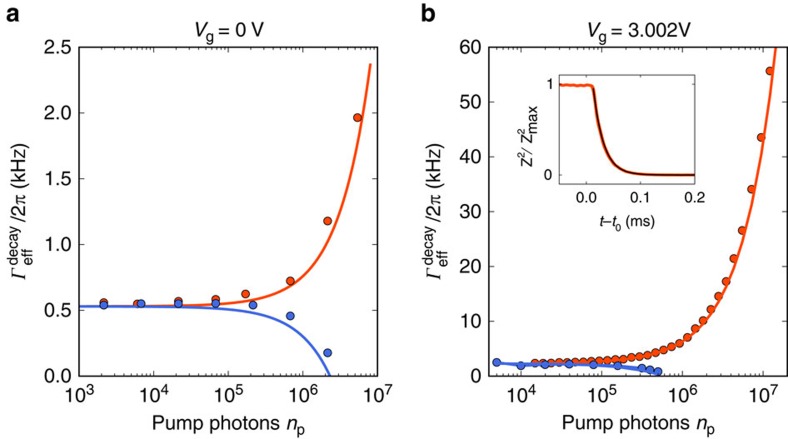
Effective mechanical energy decay rates extracted from ring-down measurements. Mechanical dissipation rate 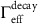 measured on device A with the ring-down technique as a function of the number *n*_p_ of pump photons in the cavity at *V*_g_=0 V and *V*_g_=3.002 V, where *n*_p_ is proportional to the microwave power *P*_in_ applied at the input of the cryostat (see Supplementary Note 3). Red and blue data points correspond to red and blue detuned pumping, respectively. The measurements are well described by 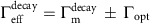 (red and blue lines) using *g*_0_/2*π*=9.7 Hz in **a** and *g*_0_/2*π*=42.6 Hz in **b**. The inset in **b** shows a ring-down measurement for *n*_p_=1.4·10^6^. We plot the normalized vibration amplitude as a function of time *t*. The resonator is driven with a capacitive driving force for *t*<*t*_0_. At *t*_0_ the drive is switched off and the vibration amplitude decays freely (*t*>*t*_0_). We fit the data with an exponential decay (black line) using 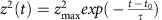 with a decay rate 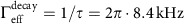. The vibration amplitude in ring-down measurements is larger than that in undriven displacement spectra, so that the motion in ring-down measurements can be resolved with lower *n*_p_.

We characterize the single-photon optomechanical coupling and show that the coupling can be significantly enhanced by deflecting the membrane towards the cavity electrode. For this, we quantify the optomechanical scattering rate *Γ*_opt_ using ring-down measurements at *V*_g_=0 V and *V*_g_=3.002 V for device A. Figure 3a,b shows the measured dissipation rate 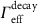 as a function of cavity pump photon number *n*_p_ for blue and red detuned pumping. The measurements are well described by 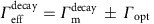 where 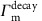 corresponds to the intrinsic mechanical dissipation rate, and ± to red and blue detuned pumping at *ω*_p_=*ω*_c_∓*ω*_m_, respectively. By increasing *V*_g_ from 0 to 3.002 V we obtain a strong increase of the optomechanical coupling from *g*_0_=2*π* × 9.7 Hz to *g*_0_=2*π* × 42.6 Hz. We estimate that the separation *d* between the membrane and the cavity counter electrode is reduced from 88 to 33 nm when varying *V*_g_ from 0 to 3.002 V. The calibration of both *g*_0_ and *n*_p_ is robust, while the quantification of the reduction of *d* is approximative; see Supplementary Notes 1 and 3, Supplementary Fig. 2 and Supplementary equations (1), (3)–(5).

### Thermal calibration and sideband cooling

**Figure 4:**
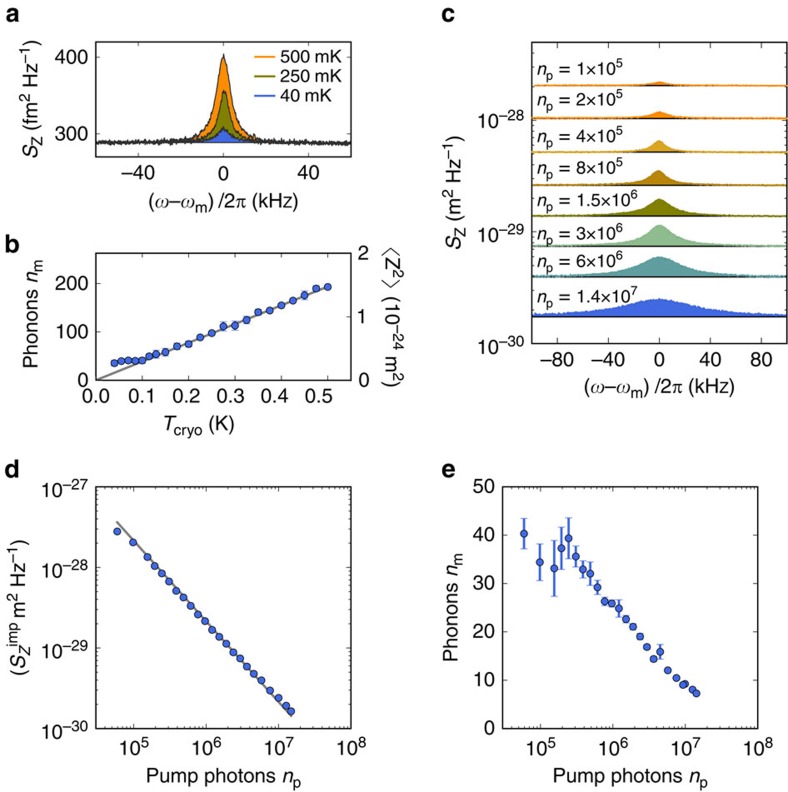
Thermal calibration and sideband cooling of fundamental mechanical mode with red-detuned pumping. (**a**) Selected thermo-mechanical noise spectra for different temperatures and *n*_p_=6·10^4^. (**b**) Plot of the measured mechanical mode temperature of device A, expressed in phonon occupation *n*_m_, as a function of cryostat temperature at *V*_g_=3.002 V where *ω*_m_/2*π*=53.7 MHz and *n*_p_=6·10^4^. On the right y-axis, we display the variance of the vibration amplitude 〈*z*^2^〉, which is obtained by integrating the thermal resonance, as is shown in **a**. The phonon occupation is quantified with 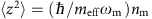 (see Supplementary Note 3). The error bars are given by the standard deviation of 5 spectral measurements. (**c**) Mechanical displacement spectral density *S*_z_ measured for different pump photon number. The cryostat temperature is 15 mK. Note that the curves are not offset. (**d**) Displacement imprecision as a function of cavity pump photon population. The line is a fit of equation (3) with *n*_add_=32. (**e**) Average phonon number *n*_m_ as a function of *n*_p_. The error bars are given by the standard deviation of five spectral measurements.

In order to calibrate the mechanical phonon occupation and the mode temperature *T*_mode_, we measure the mechanical thermal motion spectrum while varying the cryostat temperature^40^. This is done by pumping the cavity with a weak pump tone on the red sideband. The integrated area of the thermal resonance is proportional to the mode temperature according to the equipartition theorem. For temperatures above 100 mK the area is linearly proportional to the cryostat temperature, showing that the mode is in thermal equilibrium with the cryostat (Fig. 4b). This linear dependence serves as a precise calibration to relate the resonance area to the averaged phonon occupation *n*_m_ and the mode temperature *T*_mode_. Below 100 mK the mechanical mode does not thermalize well with the cryostat. The origin of this poor thermalization at low temperature may be related to the heating induced by the pump field (see below)^28^, and a non-thermal force noise^50^ such as the electrostatic force noise related to the voltage noise in the device. As a next characterization step, we investigate the mechanical phonon occupation when increasing the power of the pump tone on the red sideband and keeping the temperature of the cryostat constant at *T*_cryo_=15 mK. The measured resonance gets broader and its area smaller (Fig. 4c), showing that the mechanical mode is damped and cooled^38^,^39^. At the largest available pump power, the phonon occupation reaches *n*_m_=7.2±0.2 (Fig. 4e). This is the lowest phonon occupation reached in a mechanical resonator based on graphene^27^,^28^,^51^. The error in the estimation of *n*_*m*_ is given by the standard error obtained from five successive spectral measurements.

### Displacement sensitivity and force sensitivity

The improved coupling allows us to achieve also an excellent displacement sensitivity 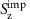 (Fig. 4d). At the largest pump power, we obtain 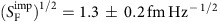, which compares favourably to previous works^27^,^51^,^52^. The error in 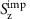 is given by the uncertainty in the estimation of *m*_eff_. We obtain 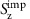 from the noise floor of the measured power spectral density *S*_N_ using 
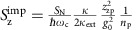
 with 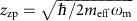 the zero-point motion amplitude^27^. The displacement sensitivity scales as 1/*n*_p_ (Fig. 4d). By comparing the measurement to the expected displacement sensitivity


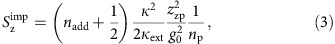


we obtain that the equivalent noise added by the amplifier chain is *n*_add_=32. This is a reasonable value for a high electron-mobility-transistor amplifier mounted at 3 K^42^,^53^.

**Figure 5:**
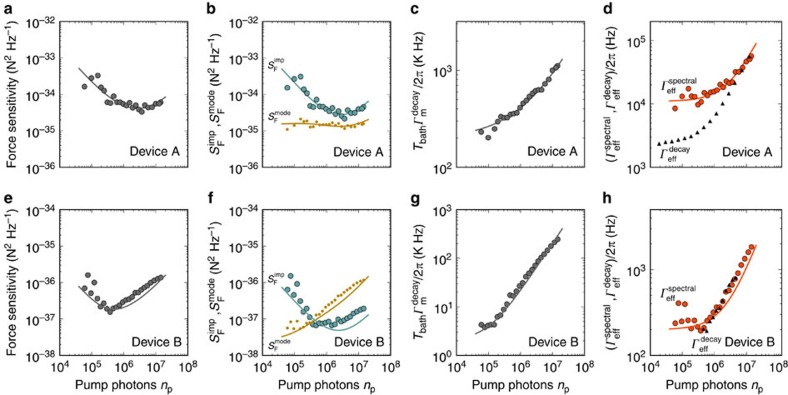
Characterization of the force sensitivity. (**a**) Force sensitivity 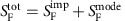 as a function of cavity pump photon population measured when pumping the cavity on the red sideband. (**b**) Imprecision force noise 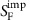 (turquoise) and thermal force noise 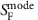 (dark yellow) versus *n*_p_. The data in **a**,**b** are fitted to equations (2), (6). (**c**) Product of the bath temperature *T*_bath_ and the intrinsic mechanical decay rate 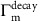 as a function of cavity pump photon occupation. The line is a fit to the data. (**d**) Effective spectral mechanical line width 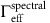 and energy decay 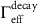 as a function of *n*_p_. The data are fitted to 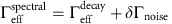 with *δ*Γ_noise_/2*π*=8.7 kHz (red line). (**e**–**h**) Equivalent to (**a**–**d**) but for device B. The lowest value for the force sensitivity in **e** is 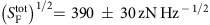. In **e** and **f** the data are fitted with *n*_add_=22 and in **h** we use *g*_0_/2*π*=7.3 Hz, *κ*/2*π*=2.5 MHz and *δ*Γ_noise_/2*π*=0.145 kHz. All the measurements on device A are performed at *V*_g_=3.002 V and on device B at *V*_g_=0 V. The cryostat temperature is 15 mK.

We now quantify the force sensitivity as a function of the microwave pump power (Fig. 5a,e). Since the mechanical resonances in the measured displacement spectra are well described by Lorentzian line shapes, the thermal force noise is quantified using 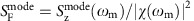 with the effective mechanical susceptibility 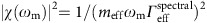. Similarly, we obtain the imprecision force noise with 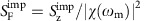. The best force sensitivity we achieve for device A is 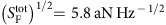 with a mechanical bandwidth of 20 kHz (Fig. 5a,d). In device B we reach a force sensitivity of 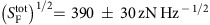 with a mechanical bandwidth of 0.2 kHz (see Fig. 5e,h). The error in the estimation of the force sensitivity is obtained from both the uncertainty in the mass and the fluctuations in the measurement of 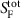, which we evaluate by calculating the standard error of 10 measurements. This force sensitivity compares favourably with the best sensitivities obtained with micro-fabricated resonators (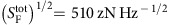)^42^,^45^, albeit it is not as good as that of resonators based on carbon nanotubes^8^,^10^. Compared to previous devices, the mechanical bandwidth of graphene resonators is much higher, which enables faster detection of sudden force changes.

## Discussion

We plot both 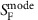 and 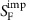 as a function of cavity pump photon population in Fig. 5b. As expected, the imprecision force noise decreases at low *n*_p_ and increases at high *n*_p_ due to the enhanced damping caused by the optomechanical back-action. The thermal force noise 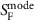 appears roughly constant when varying *n*_p_ as a result of the competing effects of Joule heating and frequency noise. Joule heating is caused by the microwave current in the graphene flake induced by the pump field. This results in the increase of the temperature *T*_bath_ of the thermal bath coupled to the mechanical mode as well as the mechanical dissipation rate^26^,^28^. We can infer the product 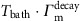 from the measurements of *n*_m_ and 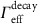 in Figs 3b and 4e using


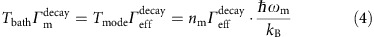


When increasing the pump power, Joule heating significantly increases the product 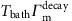 (Fig. 5c), and therefore the size of the thermal force noise (equation (1)). We see next that the effect of frequency noise leads to the opposite dependence of the thermal force noise on pump power. Frequency noise enhances the spectral line width by the amount *δΓ*_noise_,


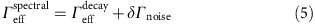


when the fluctuations of the resonant frequency are described by a white noise^8^. The measurements of 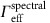 and 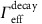 as a function of pump power can be well described by equation (5) with *δΓ*_noise_/2*π*=8.7 kHz (Fig. 5d). Importantly, Fig. 5d shows that 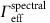 is comparable to 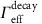 at large pump power, showing that the relative contribution of *δΓ*_noise_ to 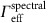 gets negligible upon increasing *n*_p_. As the cooling efficiency described by equation (4) remains unaltered by frequency noise (see chapter 7 in^54^), the thermal force noise is quantified by


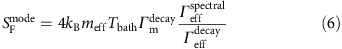


Taking into account the measured effects of Joule heating and frequency noise in equation (6), the thermal force noise 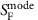 is expected to remain roughly constant as a function of *n*_p_ (dark yellow line in Fig. 5b), in agreement with the measurements. Overall, the best force sensitivity we achieve in this device is 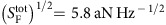 at *n*_p_≈4·10^6^ (Fig. 5a). While the force sensitivity in this device is primarily limited by the measurement imprecision, the thermal force noise is affected to a large extent by frequency noise at low *n*_p_ and by Joule heating at high *n*_p_.

In device B, the graphene resonator has a lower mass and a narrower mechanical line width, two assets for high force sensitivity (Fig. 5e–h). The spectral line width corresponds to a mechanical quality factor of *Q*≈200,000. In this device we reach a force sensitivity of 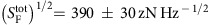 at *n*_p_≈4·10^5^ (see Fig. 5e). In an attempt to improve the thermal anchoring of device B compared to device A, the graphene contact electrodes contain an additional Au layer between the graphene and the Nb layer^28^,^55^. The normal metal layer is expected to increase the thermal conductance between the graphene flake and the contact electrodes through electron diffusion, which allows for better heat dissipation into the contacts. However, device B is still strongly affected by Joule heating, which substantially increases the value of 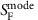 when increasing the pump power (Fig. 5f,g). The heating is so strong that we are not able to reduce the phonon occupation *n*_m_ with sideband cooling. We attribute the strong heating to the fact that the resonator is significantly thinner than the one of device A and therefore has a smaller heat capacity. The effect of frequency noise on the spectral line width is negligible for pump powers above *n*_p_≈4·10^5^. We do not know the origin of the frequency noise but it might be related to charged two-level fluctuators in the device. The force sensitivity is here primarily limited by the measurement imprecision at low *n*_p_, and by the thermo-mechanical force noise and Joule heating at high *n*_p_.

In the future, the force sensitivity of graphene optomechanical devices can be further improved using a quantum-limited Josephson parametric amplifier^53^. This readout will improve the measurement imprecision, by lowering *n*_add_ in 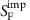. In addition, it will be possible to resolve the thermal vibrations with lower pump power, which is crucial to reduce Joule heating while working with low-mass graphene resonators. A quantum-limited amplifier with *n*_add_=0.5 may allow to achieve 47 zN Hz^−1/2^ force sensitivity at 15 mK taking the mass of a single-layer graphene resonator with the diameter and the quality factor of device B (Fig. 1b). With only modest device improvements, it may be possible to probe the fundamental limit of continuous displacement detection imposed by quantum mechanics, since the force noise associated to quantum backaction 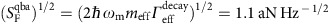 is approaching 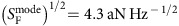 measured at *n*_p_=1.4 × 10^7^ for device A. Force sensing with resonators based on two-dimensional materials hold promise for detecting electron and nuclear spins^33^ using superconducting cavities compatible with relatively large magnetic fields^56^, and studying the thermodynamic properties of two-dimensional materials, such as the quantum capacitance and the magnetization^35^.

## Methods

### Cavity characterization

In Fig. 2d,e we plot the coefficient |*S*_11_|^2^ and the phase of the reflected signal when sweeping the frequency over the cavity resonance at *ω*_c_/2*π*=7.416 GHz. To extract the external coupling rate *κ*_ext_ and the internal loss rate *κ*_int_ we fit the measurement with the line shape expected for a one-port reflection cavity^57^


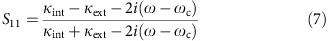


which yields *κ*_int_/2*π*=950 kHz and *κ*_ext_/2*π*=850 kHz at *V*_g_=3.002 V for device A. The rates of Device B are *κ*_int_/2*π*=800 kHz and *κ*_ext_/2*π*=1700  kHz at *V*_g_=0 V.

### Data availability

The data that support the findings of this study are available from the corresponding author upon request.

## Additional information

**How to cite this article:** Weber P. *et al*. Force sensitivity of multilayer graphene optomechanical devices. *Nat. Commun.* 7:12496 doi: 10.1038/ncomms12496 (2016).

## Supplementary Material

Supplementary Information Supplementary Figures 1 & 2, Supplementary Notes 1-3, Supplementary References
