# Supplementary material for: Force sensitivity of multilayer graphene optomechanical devices
Source: Nat Commun. 2016 Aug 9;7:12496. doi: 10.1038/ncomms12496 (PMC4980493; doi:10.1038/ncomms12496)
Supplement: Supplementary Information — Supplementary Figures 1 & 2, Supplementary Notes 1-3, Supplementary References [file ncomms12496-s1.pdf]

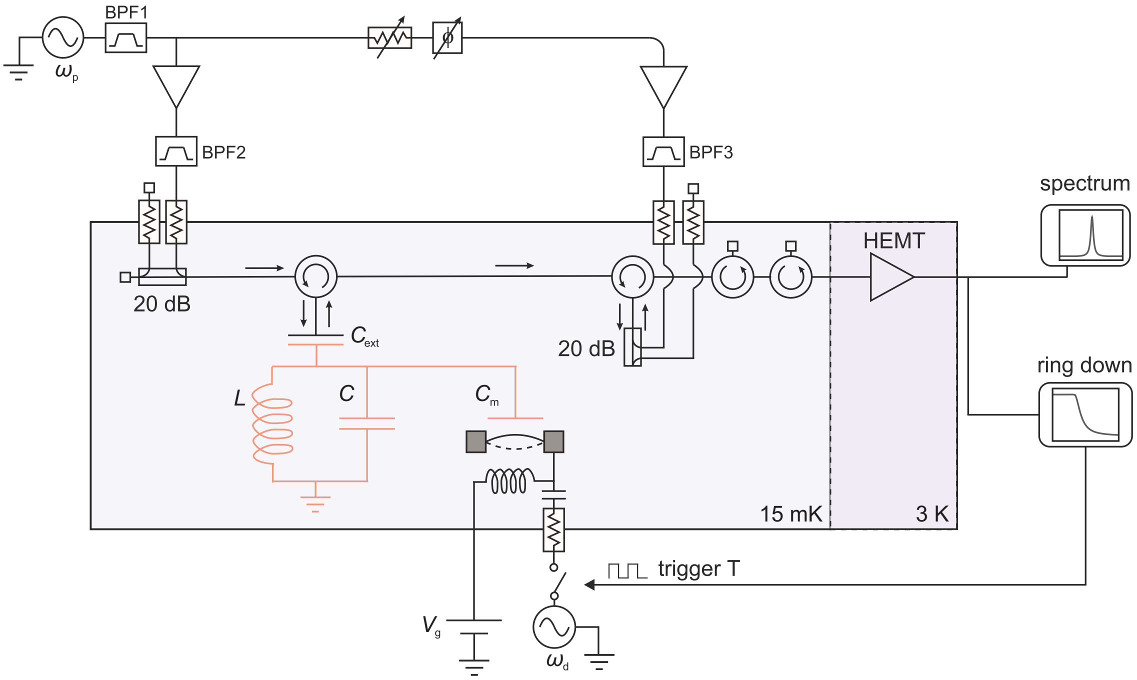

Supplementary Figure 1: Measurement setup with cryogenic wiring. See Supplementary Note 2 for details.

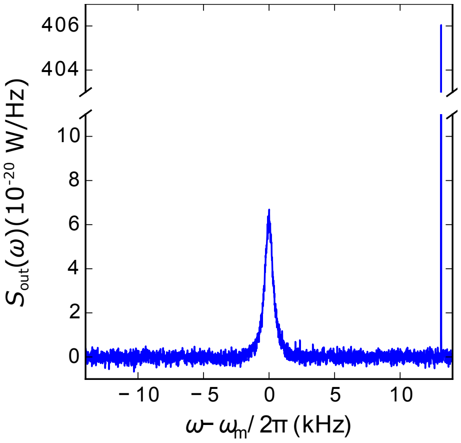

Supplementary Figure 2: Calibration of single-photon optomechanical coupling. Output spectrum of mechanical sideband and FM signal at $T = 220\,\mathrm{mK}$ for device B. The mechanical resonance frequency is $\omega_\mathrm{m}/2\pi = 46.06\,\mathrm{MHz}$ and the modulation frequency $\omega_\mathrm{mod}/2\pi = \omega_\mathrm{m}/2\pi + 13.1\,\mathrm{kHz}$. The data is taken in a regime where backaction is negligible, $n_\mathrm{p} = 9 \cdot 10^4$. The modulation depth ϕ_0 is measured by extracting the ratio between pump signal and the first sideband signal $S_\mathrm{out}(\omega_\mathrm{p} + \omega_\mathrm{out})/S_\mathrm{out}(\omega_\mathrm{p}) = \phi_0/2$ for $\phi_0 << 1$. We use $\phi_0 = 10^{-116.7\,dB/20} \cdot 2 = 2.9244 \cdot 10^{-6}$ for this measurement. Note that we have subtracted the measurement imprecision in the figure. See supplementary Note 3 for details.

SUPPLEMENTARY NOTE 1. MECHANICAL PROPERTIES OF THE GRAPHENE MEMBRANE

Static deflection

The deflection of the graphene flake of device A at $V_\mathrm{g} = 3.002\,\mathrm{V}$ can be quantified approximately. We assume that the shape of the static graphene deformation depends on the radial coordinate r as $\xi_\mathrm{s}(r) = z_\mathrm{s}(1 - r^2/R^2)$ where z_s is the deflection of the center point of the circular membrane and R its radius [1]. With this deflection profile we evaluate the derivative of the capacitance with respect to displacement using a local plate capacitor approximation

$$\partial_\mathrm{z} C_\mathrm{m} = \epsilon_0 \int_0^{2\pi} d\phi \int_0^{R_\mathrm{g}} r dr \frac{\partial}{\partial z_\mathrm{s}} \frac{1}{d_0 - z_\mathrm{s}(1 - \frac{r^2}{R^2})}. \qquad \text{Supplementary Equation 1}$$

Here R_g is the radius of the cavity counterelctrode. Using the measured ω_m and g_0 values (Figs. 2h and 3a,b, main text) in $g_0 \propto \partial_\mathrm{z} C_\mathrm{m} \sqrt{1/\omega_\mathrm{m}}$ [1], we estimate that the separation between the membrane and the cavity counter electrode is reduced from 88 nm to 33 nm when varying V_g from 0 to 3.002 V.

Mechanical resonance frequency

Deflecting the graphene membrane by tuning $|V_\mathrm{g}|$ results in the reduction of both ω_c and ω_m (Figs. 2g,h, main text). The reduction of $\omega_\mathrm{m}/2\pi$ is particularly large as it decreases from 67 MHz at $V_\mathrm{g} = 0\,\mathrm{V}$ to below 40 MHz at $V_\mathrm{g} = 3.055\,\mathrm{V}$ for device A. This decrease is attributed to the electrostatic softening of the graphene spring constant [2, 3]. For a critical V_g, the electrostatic force overwhelms the mechanical restoring force, ω_m drops to zero, and the resonator collapses against the cavity counter electrode. From the comparison between the measurement and the expected electrostatic softening for weak resonator deflection,

$$\omega_\mathrm{m} = \sqrt{\omega_{\mathrm{m},0} - \frac{1}{2m_\mathrm{eff}} (\partial_\mathrm{z}^2 C_\mathrm{m}) V_\mathrm{g}^2} \qquad \text{Supplementary Equation 2}$$

with $\partial_\mathrm{z}^2 C_\mathrm{m}$ obtained by differentiating Supplementary Equation 1, we get $m_\mathrm{eff} = 3.3 \cdot 10^{-17}\,\mathrm{kg}$ (solid line of Fig. 2h, main text). This value differs from $m_\mathrm{eff} = 4.9 \cdot 10^{-17}\,\mathrm{kg}$ estimated from the dimensions of the resonator measured by AFM. Both methods may lead to an error in the estimation of m_eff, as AFM in air can overestimate the graphene thickness due to electrostatic forces induced by

humidity adsorbed on the surface [4], while the mass estimated from electrostatic softening depends on the assumed mode shape, which is not well known. Therefore, we use $m_{\text{eff}} = (4.1 \pm 0.8) \cdot 10^{-17}$ kg for device A, which corresponds to approximately 25 layers. For device B we obtain consistently $5-6$ layers, both by measuring the dependence of ω_{m} on V_{g} and by optical contrast measurements [5, 6].

SUPPLEMENTARY NOTE 2. MEASUREMENT SETUP

Supplementary Figure 1 shows a detailed schematic of the measurement setup. We perform the measurements in a Triton 200 dilution refrigerator from Oxford instruments with a base temperature of 15 mK. The RF-lines are UT85-SS-SS coaxial cables from room temperature to the 700 mK stage and superconducting UT85-Nb-Nb coaxial cables from 700 to 15 mK. The input lines are attenuated by cryogenic attenuators to decrease the thermal electromagnetic noise from room temperature. The attenuation is 10 dB at $T = 47$ K, 20 dB at $T = 3$ K, 6 dB at $T = 700$ mK. At $T = 15$ mK we use an Arra 5191-20 20 dB directional coupler on the pump line and a Krytar 120420 20 dB directional coupler on the cancellation line; for details on cancellation see below. The circulators are CTH0408KC from Quinstar. We use two circulators at the output line to shield the sample from the thermal noise of the 3 K stage.

The source used to apply the constant voltage V_{g} is a SIM928 from Stanford Research Systems. The DC line is filtered with π-filters at room temperature and a custom made RC filter at the mixing chamber. A bias tee ZFBT-6GW from minicircuits connects the V_{g} line with the coaxial cable used to apply the low frequency drive. In contrast to the directional couplers employed for the thermalisation of the pump and cancellation lines, we use a 20 dB cryogenic attenuator at 15 mK on the drive line.

The cavity pump tone at frequency ω_{p} is generated with an Agilent E8257D PSG microwave source. An Agilent N5181A microwave source is used to generate the MHz frequency drive at ω_{d}. We divide the signal of the pump source with a power splitter into a cavity pump signal and a cancellation signal. The digitally variable attenuator TEA13000-12 and variable phase shifter TEP8000-6 from Telemakus cancel the pump tone by ≈ 40 dB to avoid saturation of the cryogenic high-electron-mobility transistor (HEMT) amplifier. The HEMT is a LNF-LNC4_8A from Low Noise Factory.

We amplify and filter the pump and the cancellation signals at room temperature. Careful filtering of the input signal is necessary to avoid populating the cavity with source phase noise or

with noise of the amplifier. For this we use tunable bandpass filters. The bandpass filter BPF1 (Wainwright WBCQV6) at the output of the pump source has a pass band attenuation of $-6\,\mathrm{dB}$ and reduces the phase noise of the pump signal by $-50\,\mathrm{dB}$ at $\pm 50\,\mathrm{MHz}$ from its pass band frequency. The amplification is done with Mini-circuits ZVE-3W-83+ amplifiers. We reduce the amplifier white noise by a second set of bandpass filters BPF2 and BPF3 (both wainwright WTBCQV3). They attenuate the signal by $-2\,\mathrm{dB}$ on the pass band frequency range and by $-20\,\mathrm{dB}$ at $\pm 50\,\mathrm{MHz}$ away from it. Without this filtering scheme we populate the superconducting cavity at the highest pump powers.

We characterize the properties of the superconducting cavity with a vector-network analyzer (VNA) ZVB14 from Rohde&Schwarz. For spectral measurements we use an Agilent MXA N9020A with integrated preamplifier.

SUPPLEMENTARY NOTE 3. OPTOMECHANICAL DEVICE PROPERTIES

In order to calibrate the phonon occupation n_m, the single-photon optomechanical coupling g_0, and the cavity pump photon number n_p, we perform the following three characterization measurements on both devices. (i) The measurement of the thermal resonance as a function of cryostat temperature T_cryo. This allows for the calibration of n_m by relating the integrated area of the thermal resonance to T_cryo. (ii) The measured transmitted power between the input port and the output port of the cryostat several MHz away from the cavity resonance frequency, which quantifies the product of $loss \cdot gain$. Here $loss$ is the effective loss coefficient along the input line and $gain$ is the effective gain coefficient along the output line. The product $loss \cdot gain$ together with the measurement in (i) yield g_0. (iii) Knowing g_0, the measured mechanical decay rate of the graphene resonator as a function of the pump power applied at the input port of the cryostat allows us to calibrate n_p. We discuss this procedure in detail further below.

We additionally check with device B that this calibration procedure yields the same g_0 as that obtained with the procedure based on the frequency modulation (FM) of the pump field [7, 8]. In the latter, g_0 is inferred by pumping the cavity with a FM signal with known modulation index ϕ_0 and modulation frequency $\omega_\mathrm{mod}/2\pi$, see Supplementary Figure 2. The modulation frequency is chosen close to the mechanical frequency, but outside the mechanical line width. When applying the FM signal additionally to the red sideband pump, the single-photon optomechanical coupling can be extracted by measuring the ratio of the area under the mechanical sideband to the area under the modulation peak using

$$g_0^2 = \left(\frac{\phi_0}{2}\right)^2 \frac{\omega_\mathrm{m}^2}{n_\mathrm{m}} \frac{S_\mathrm{out}(\omega_\mathrm{m}) \cdot \mathit{\Gamma}_\mathrm{m}/4}{S_\mathrm{out}(\omega_\mathrm{mod}) \cdot RBW}. \qquad \text{Supplementary Equation 3}$$

Here, S_out is the power spectral density at the output of the cryostat, and $\mathit{\Gamma}_\mathrm{m}$ and RBW are the mechanical line width and the measurement resolution band width, respectively. Both $\mathit{\Gamma}_\mathrm{m}$ and RBW are taken in units of frequency. Performing this analysis in thermal equilibrium at $T = 220\,\mathrm{mK}$ we obtain $g_0/2\pi = 8.4\,\mathrm{Hz}$, which is in agreement with the value $g_0/2\pi = 7.3\,\mathrm{Hz}$ obtained from the first calibration method discussed in this section.

Mechanical mode occupation

The integrated area of the thermal resonance is a direct probe of the temperature of the mechanical mode and therefore allows for the calibration of the mechanical phonon occupation [9]. In Fig. 4b of the main text we show a measurement of the integrated area, represented in units of n_m, of the mechanical thermal noise spectra versus cryostat temperature at $V_\mathrm{g} = 3.002\,\mathrm{V}$ for device A. We pump the cavity on the red sideband with a weak pump tone (so that the optomechanical scattering rate $\mathit{\Gamma}_\mathrm{opt}$ is much smaller than the intrinsic dissipation rate $\mathit{\Gamma}_\mathrm{m}^\mathrm{decay}$ of the graphene resonator), and measure the area of the thermal mechanical resonance at ω_c. In thermal equilibrium, the area is linearly proportional to the motional variance $\langle z^2 \rangle$ and thus directly linked to the temperature of the mode T_mode through the equipartition theorem $1/2 m_\mathrm{eff}\omega_\mathrm{m}^2 \langle z^2 \rangle = 1/2 k_\mathrm{B} T_\mathrm{mode}$. In Fig. 4b of the main text, T_mode is linearly proportional to T_cryo for temperatures above 100 mK such that we assign $T_\mathrm{mode} = T_\mathrm{cryo}$ and extract the resonator phonon occupation $n_\mathrm{m} = \frac{k_\mathrm{B} T_\mathrm{mode}}{\hbar\omega_\mathrm{m}}$ with ω_m the angular frequency of the mechanical resonator.

Single-photon optomechanical coupling

The measurement described in the previous subsection also allows to accurately calibrate g_0. It is inferred from the area of the thermal resonance measured as the output power $P_\mathrm{out} = \int S_\mathrm{out} d\omega/2\pi$ at the level of the spectrum analyzer and the input power P_in applied at the input port of the cryostat. For all available pump powers, we carefully calibrate the power P_in as a function of the power P_RF applied with the radio-frequency source. The ratio $P_\mathrm{out}/P_\mathrm{in}$ for a reflection cavity reads [1, 7, 10]

$$\frac{P_\mathrm{out}}{P_\mathrm{in}} = 4 g_0^2 \cdot gain \cdot loss \cdot \frac{\kappa_\mathrm{ext}^2}{\kappa^2} \frac{1}{\omega_\mathrm{m}^2} \frac{k_\mathrm{B}}{\hbar\omega_\mathrm{m}} T_\mathrm{cryo}. \qquad \text{Supplementary Equation 4}$$

We determine the product $loss \cdot gain = -32.1\,\mathrm{dB}$ by measuring the transmitted power between the input port and the output port of the cryostat at a frequency $\omega_\mathrm{p} = \omega_\mathrm{c} - \omega_\mathrm{m}$. From the data shown in Fig. 4b we then extract $g_0 = 2\pi \times 42.6\,\mathrm{Hz}$, which is the largest value achieved so far with graphene optomechanical systems [7, 9]. For this calibration to be precise, we verify that the response of the transmitted power through our input and output lines remains constant over the frequency range between ω_c and ω_p.

Calibration of cavity pump photon number

Knowing g_0 and κ at $V_\mathrm{g} = 3.002\,\mathrm{V}$, we are now able to relate P_in to n_p. To do so we measure the mechanical decay rate with the ring-down technique as a function of P_in and fit the data with $\Gamma_\mathrm{eff}^\mathrm{decay} = \Gamma_\mathrm{m}^\mathrm{decay} \pm 4 n_\mathrm{p} g_0^2/\kappa$ (see Fig. 3b, main text). This allows us to estimate $loss = -63.7\,\mathrm{dB}$ using [11]

$$n_\mathrm{p} = \frac{1}{\hbar\omega_\mathrm{p}} \cdot P_\mathrm{in} \cdot loss(\omega_\mathrm{p}) \cdot \frac{4\kappa_\mathrm{ext}}{\kappa^2 + 4(\omega_\mathrm{p} - \omega_\mathrm{c})^2}. \qquad \text{Supplementary Equation 5}$$

From the value of $loss$ and the product $gain \cdot loss$, we obtain $gain = 31.6\,\mathrm{dB}$. The increase of $\Gamma_\mathrm{m}^\mathrm{decay}$ due to heating by the pump photons is modest compared to Γ_opt at large n_p, so that it has no influence on the estimation of $loss$. In order to get g_0 at $V_\mathrm{g} = 0\,\mathrm{V}$, we use the gate voltage independent calibration of n_p to fit the measured n_p dependence of $\Gamma_\mathrm{eff}^\mathrm{decay}$ in Fig. 3a of the main text. We obtain $g_0 = 2\pi \times 9.7\,\mathrm{Hz}$. For device B we perform the calibration in the same manner and obtain $g_0/2\pi = 7.3\,\mathrm{Hz}$ at $V_\mathrm{g} = 0\,\mathrm{V}$.

SUPPLEMENTARY REFERENCES

---

[1] Weber, P., Güttinger, J., Tsioutsios, I., Chang, D. E., and Bachtold, A. Coupling graphene mechanical resonators to superconducting microwave cavities, Nano Letters **14**, 2854-2860 (2014).

[2] Eichler, A., Moser, J., Chaste, J., Zdrojek, M., Wilson-Rae, I., and Bachtold, A. Nonlinear damping in mechanical resonators made from carbon nanotubes and graphene, Nat. Nanotech. **6**, 339-342 (2011).

[3] Kozinsky, I., Postma, H. W. Ch., Bargatin, I., and Roukes, M. L. Tuning nonlinearity, dynamic range, and frequency of nanomechanical resonators, Appl. Phys. Lett. **88**, 253101 (2006).

[4] Moser, J., Verdaguer, A., Jimenez, D., Barreiro, A., and Bachtold, A. The environment of graphene probed by electrostatic force microscopy, Appl. Phys. Lett. **92**, 123507 (2008).

[5] Blake, P. et al. Making graphene visible, Appl. Phys. Lett. **91**, 063124 (2007).

[6] Ni, Z. H. et al. Graphene Thickness Determination Using Reflection and Contrast Spectroscopy, Nano Letters **7**, 2758-2763 (2007).

[7] Singh, V., Bosman, S. J., Schneider, B. H., Blanter, Y. M., Castellanos-Gomez, A., and Steele, G. A. Optomechanical coupling between a multilayer graphene mechanical resonator and a superconducting microwave cavity, Nat. Nanotech. **9**, 820-824 (2014).

[8] Zhou, X. et al. Slowing, advancing and switching of microwave signals using circuit nanoelectromechanics, Nat. Phys. **9**, 179-184 (2013).

[9] Song, X., Oksanen, M., Li, J., Hakonen, P. J., and Sillanpää, M. A. Graphene optomechanics realized at microwave frequencies, Phys. Rev. Lett. **113**, 027404 (2014).

[10] Hertzberg, J. B., Rocheleau, T., Ndukum, T., Savva, M., Clerk, A. A., and Schwab, K. C. Back-action-evading measurements of nanomechanical motion, Nat. Phys. **6**, 213-217 (2010).

[11] Aspelmeyer, M., Kippenberg, T. J., and Marquardt, F. Cavity Optomechanics, Rev. Mod. Phys **86**, 1391 (2014).
